# Cost-effectiveness of various immunization schedules with inactivated Sabin strain polio vaccine in Hangzhou, China

**DOI:** 10.3389/fpubh.2022.990042

**Published:** 2022-09-23

**Authors:** Yuyang Xu, Yan Liu, Jun Wang, Xinren Che, Jian Du, Xiaoping Zhang, Wenwen Gu, Xuechao Zhang, Wei Jiang

**Affiliations:** Department of Expanded Program on Immunization, Hangzhou Center for Disease Control and Prevention, Hangzhou, China

**Keywords:** cost-effectiveness, dose, IPV, immunization schedules, Sabin strain

## Abstract

**Background:**

It is necessary to select suitable inactivated poliovirus vaccine(IPV) and live, attenuated oral poliovirus vaccine (OPV) sequential immunization programs and configure the corresponding health resources. An economic evaluation was conducted on the sequential procedures of Sabin strain-based IPV (sIPV) and bivalent OPV (bOPV) with different doses to verify whether a cost-effectiveness target can be achieved. This study aimed to evaluate the cost-effectiveness of different sIPV immunization schedules, which would provide convincing evidence to further change the poliovirus vaccine (PV) immunization strategies in China.

**Methods:**

Five strategies were included in this analysis. Based on Strategy 0(S_0_), the incremental cost (IC), incremental effect (IE), and incremental cost-effectiveness ratio (ICER) of the four different strategies (S_1_/S_2_/S_3_/S_4_) were calculated based on the perspective of the society. Seven cost items were included in this study. Results of field investigations and expert consultations were used to calculate these costs.

**Results:**

The ICs of S_1_/S_2_/S_3_/S_4_ was Chinese Yuan (CNY) 30.77, 68.58, 103.82, and 219.82 million, respectively. The IE of vaccine-associated paralytic poliomyelitis (IE_VAPP_) cases of S_1_/S_2_/S_3_/S_4_ were 0.22, 0.22, 0.22, and 0.11, respectively, while the IE of disability-adjusted life-years (IE_DALY_) of S_1_/S_2_/S_3_/S_4_ were 8.98, 8.98, 8.98, and 4.49, respectively. The ICER_VAPP_ of S_1_/S_2_/S_3_/S_4_ gradually increased to CNY 13.99, 31.17, 47.19, and 199.83 million/VAPP, respectively. The ICER_DALY_ of S_1_/S_2_/S_3_/S_4_ also gradually increased to CNY 0.34, 0.76, 1.16, and 4.90 million/DALY, respectively.

**Conclusion:**

ICER_VAPP_ and ICER_DALY_ were substantially higher for S_3_ (four-sIPV) and S_4_ (replacement of self-funded sIPV based on one-sIPV-three-bOPV). Two-sIPV-two-bOPV had a cost-effectiveness advantage, whereas S2/S3/S4 had no cost-effectiveness advantage.

## Introduction

Poliovirus can cause limb paralysis, an acute intestinal tract infectious disease commonly known as poliomyelitis. To date, there is no effective treatment for poliomyelitis, and it can only be prevented through vaccination (1). There are three serotypes of poliovirus including serotypes I, II, and III, all of which can cause acute infectious diseases without cross-immunity (2). There are two types of poliovirus vaccines (PV): live, attenuated oral poliovirus vaccine (OPV) and inactivated poliovirus vaccine (IPV). OPV was produced using Sabin virus strains, which were divided into trivalent OPV (tOPV), bivalent OPV (bOPV), and monovalent OPV (mOPV), according to the strains contained in the vaccine. TOPV contains type I, II, and III virus strains, while bOPV contains type I and III virus strains; mOPV is divided into type I-mOPV, type II-mOPV, and type III-mOPV. OPV was first licensed as monovalent OPV (mOPV) in 1961 and trivalent OPV (tOPV) in 1963. IPV was made available in the market and licensed in 1955 (2). OPV is a safe and effective vaccine and has become the vaccine of choice for the Global Polio Eradication Initiative (GPEI) owing to its high immunization efficacy, low cost, and convenient administration. The extremely effective programs implemented by the World Health Organization (WHO) resulted in the declaration of the eradication of wild-type 2 poliovirus in 2016 and wild-type 3 poliovirus in 2019 (3). However, vaccine-associated paralytic poliomyelitis (VAPP) and vaccine-derived poliovirus (VDPV) can occur in an under-immunized population. IPV can be produced using Sabin and Salk strains, both of which are trivalent vaccines that are effective against poliovirus types I, II, and III. IPV can produce an ideal immune response and durable immunity, and inactivation of the vaccine virus does not cause VAPP and VDPV (4). However, owing to its high price, IPVs are mostly used in developed countries (5).

Since 1988, when the WHO began to carry out GPEI (6, 7), the WHO regions of the Americas, Western Pacific, and Europe were officially declared polio free (8), region of South-East Asia was certified in 2014, African region was certified in 2020 (3). China has made significant progress in polio eradication through the use of OPV, strengthening OPV routine immunization (RI) and supplementary immunization activities, and strengthening surveillance of acute flaccid paralysis (AFP). China has been free of indigenous polio cases since September 1994, when the last case of indigenous wild poliovirus (WPV) was reported. In October 2000, the WHO region of Western Pacific, including China's, was declared polio free (9). Polio eradication refers not only to the elimination of WPVs, but also to the elimination of VAPP and VDPV cases. In theory, as long as OPV is used, the above problems are inevitable. Before May 2016, China's PV immunization was implemented as follows: one dose of OPV at 2, 3, and 4 months old and one dose of OPV at 4 years old. The WHO formulated the Polio Eradication and Endgame Strategic Plan 2013–2018 (3) as well as the third Global Action Plan to minimize poliovirus facility-associated risk (GAPIII) (10) requiring all countries to use at least one dose of IPV, replace tOPV with bOPV by the end of April 2016, and finally stop using OPV in 2019–2020. In May 2016, China began to change the PV immunization strategy. Children were vaccinated with one dose of IPV at 2 months old, and one dose of bOPV at 3, 4 months, and 4 years old. On January 14, 2015, China Food and Drug Administration approved the production of the world's first inactivated vaccine (single vaccine) from Sabin polio strain produced by the Institute of Medical Biology, Chinese Academy of Medical Sciences, and the vaccine was officially put on the market in July 2015. The listing of domestic Sabin strain-based IPV (sIPV) provides the possibility for IPV to be included in China's immunization schedule, which plays a crucial role in the complete eradication of poliomyelitis in China (11). Currently, the IPV used in China's immunization planning includes sIPV produced by domestic enterprises and Salk strain-based IPV imported from abroad, and sIPV is primarily used. At present, there is shortage of sIPV supply worldwide (12), and China is one of the few countries that can produce sIPV. In addition, IPV will gradually replace OPV, and Chinese sIPV will play a major role.

Domestic sIPV demonstrated good immunogenicity and safety profile, and the positive conversion rates of neutralizing antibodies against types I, II, and III were 100, 94.9, and 99.0%, respectively, after three doses of basic immunization (13). The sequential procedures of IPV and OPV vary in different countries worldwide, and 1–4 doses of IPV and OPV are used (5). bOPV is a newly developed vaccine in China. Studies have shown that after sequential immunization with Salk strain-based IPV and domestic bOPV at one or two doses, the positive conversion rate of neutralizing antibodies against serum poliovirus types I and III can reach up to >94%, and it is safe (14). According to GAPIII, China will implement the transformation of PV immunization strategies in the future. Hence, it is necessary to select suitable IPV and OPV sequential immunization programs and configure the corresponding health resources. At the same time, the acceptance of different populations to the transformation strategy should be considered prior to the transformation to ensure the smooth implementation of the transformation of the immunization strategy and maintain a polio-free status. Adjustment of the immunization strategy of PV will also greatly affect the allocation of funds and resources for public health immunization programs. An economic evaluation is a comparative analysis of the costs and results of alternative activity processes (15). Therefore, a health economic evaluation must be conducted before adjusting the immunization strategy for PV. An economic evaluation was conducted on the sequential procedures of sIPV and bOPV with different doses to verify whether a cost-effectiveness target can be achieved. The incremental cost-effectiveness ratio (ICER), one of the commonly used evaluation indices, can be used to evaluate the relationship between two or more alternative treatment economies (16) and is suitable for the comparison of different immune programs.

## Materials and methods

### Study strategy

According to the procedures currently used in Hangzhou and those required by the WHO for conversion, five strategies were included in the analysis. Strategy 0 (S_0_): one-sIPV-three-bOPV, Strategy 1 (S_1_): two-sIPV-two-bOPV, Strategy 2 (S_2_): three-sIPV-one-bOPV, Strategy 3 (S_3_): four-sIPV, and Strategy 4 (S_4_): calculation of the replacement of the self-funded sIPV based on S_0_.

### Economic indicators

The IC, incremental effectiveness (IE), and ICER of the four different strategies were calculated based on the perspective of the society. IC refers to the difference between the cost change of the S_1_/S_2_/S_3_/S_4_ PV immunization strategy and that of S_0_. The efficacy was measured based on the incidence of VAPP and disability-adjusted life-years (DALY). The DALY is a comprehensive measure of healthy life years lost to disease-related death and disability. To calculate the DALY of VAPP, the years of life lost and years lived with disability were comprehensively calculated. The age relative value (age weight) and time relative value (discounting rate) of life-years were used for weighted adjustment. IE refers to the difference between the effect index of strategies S_1_/S_2_/S_3_/S_4_ and S_0_ and the ratio of incremental cost (IC) to IE, namely, the ICER.

The variable costs in this study included seven items: vaccine cost, vaccination cost, storage and transportation cost, medical waste disposal cost, adverse reactions related to PV, VDPV-related cost, and parents' traffic delay cost. The cost of an outbreak was not included in the analysis (17).

Field investigation and expert consultation were used to calculate the above costs.

### Incremental cost composition

#### Basic parameters

##### Number of newborns

In the Opinions of the Central Committee of the Communist Party of China and the State Council on Adjusting and Improving the Family Planning Policy issued in December 2013, the policy of allowing couples to have two children if one parent was an only child was implemented. According to the total population in the Statistical bulletin of Hangzhou Bureau of Statistics from 2013 to 2018 and the change rule of the number of newborns in Hangzhou Immunization Planning Information System, the average number of newborns from 2013 to 2018 is 123,373 (Supplementary Table 1).

##### PV immunization coverage rate

The monitoring report of the RI coverage rate can dynamically reflect the overall RI situation in a timely manner. According to a report on the RI coverage rate in Hangzhou from 2016 to 2018, the vaccination coverage rate of four doses of PV in all districts and counties exceeded 99%. To ensure the reliability of the monitoring data, Hangzhou carried out an annual field survey on the vaccination rate of school-age children, which is consistent with the routine monitoring data. The PV coverage rates of the four doses in this study were calculated and analyzed to be 99%.

##### Self-funded sIPV replacement rate

The self-funded sIPV replacement rate was defined as the proportion of self-funded sIPV (including DTaP/Hib/IPV) as replacement for free sIPV and bOPV routine vaccination. Data on the usage of self-funded sIPV and DTaP/Hib/IPV in Hangzhou from 2016 to 2018 were obtained from the annual report on non-immunization program (NIP) vaccine use in Hangzhou. Data on the PV vaccination dose in Hangzhou from 2016 to 2018 were obtained from the monthly report on RI (Supplementary Table 2). The calculated average replacement rate for self-funded sIPV from 2016 to 2018 was 36.54%. The declining trend in the self-funded sIPV replacement rate from 2016 to 2018 is mainly related to the insufficient supply of DTaP/Hib/IPV. The supply of DTaP/Hib/IPV returned to normal in 2019; therefore, the self-funded sIPV replacement rate in this study was 50%.

##### Loss coefficient

The loss coefficient of bOPV was 2.50, while that of sIPV was 1.05 based on the regulations for vaccination. According to the monthly report on the Hangzhou immunization planning vaccine, the loss coefficient of bOPV was 1.68, while that of free sIPV was 1.01 from 2016 to 2018. In this study, the bOPV loss coefficient was 1.70, the free sIPV loss coefficient was 1.01, the self-funded sIPV loss coefficient was not considered, and the loss coefficient of syringes was 1.05.

##### Immunization schedules

In this study, infants aged 2, 3, and 4 months received one dose of PV, while those aged 4 years received one booster dose. The study strategy, S_0_/S_1_/S_2_/S_3_/S_4_, was calculated as four doses of PV per child.

##### Economic indicators

The per capita gross domestic product (GDP) data used in this study were obtained from the official website of the Hangzhou Bureau of Statistics (http://tjj.hangzhou.gov.cn/), and the per capita GDP announced by the Hangzhou Bureau of Statistics in 2018 was Chinese Yuan (CNY) 140,180 per year. The CNY exchange rate data used in this study were obtained from the State Administration of Foreign Exchange (http://www.safe.gov.cn). In 2018, the exchange rate of CNY to USD was 1:6.86. This study did not discount the future, but only described the data from the literature published before 2018.

#### Composition of IC

##### Vaccine IC

Vaccine IC for S_1_/S_2_/S_3_/S_4_ = vaccine cost for S_1_/S_2_/S_3_/S_4_ – vaccine cost for S_0_.

Vaccine cost = number of newborns × PV coverage rate × (sIPV loss coefficient × sIPV dose × sIPV vaccine price + bOPV loss coefficient × bOPV dose × bOPV vaccine price).

According to the price of vaccines purchased through bidding in Zhejiang Province, free sIPV was CNY 35 per dose, bOPV was CNY 2.72 per dose, self-funded sIPV was CNY 168 per dose, and DTaP/Hib/IPV was CNY 599 per dose.

The vaccine cost of S_0_ was CNY 6011,939, while the vaccine ICs of S_1_/S_2_/S_3_/S_4_ were CNY 3,752,851, 7,505,703, 11,258,554, and 29,931,938, respectively. The costs of S_1_-S_4_ increased gradually.

##### Vaccination IC

Vaccination IC of S_1_/S_2_/S_3_/S_4_ = vaccination cost of S_1_/S_2_/S_3_/S_4_ – vaccination cost of S_0_.

Vaccination cost = number of newborns × vaccination rate of PV × [sIPV dose times × (syringe price × syringe loss coefficient + sIPV vaccination subsidy + sIPV vaccination working time cost) + bOPV dose times × (bOPV vaccination subsidy + bOPV vaccination working time cost)].

The price of the syringe in this study refers to the price of a 2-ml syringe in Zhejiang province, which is set at CNY 0.22 per syringe.

According to the principle of “Notice on Strengthening Vaccination Work” issued by the General Office of the Ministry of Health in 2012, each dose of planned immunization vaccine should not be less than CNY 5 of the subsidy. The service fee for non-immunization vaccination is CNY 28 per dose, according to the price standard of Zhejiang Province. In this study, the subsidy for free vaccine injection was CNY 5 per dose, while the subsidy for self-funded vaccine was CNY 28 per dose.

Due to time constraints, this study in the process of estimating incremental work time cost, combined with routine vaccination outpatient service supervision and assessment through the monitoring of timely sIPV and bOPV vaccination differences in work time and the vaccination that the doctors know, confirm that the differences in the influence of two vaccines' work time are not obvious, and the incremental work time cost is zero.

The IC inoculated in S_1_/S_2_/S_3_/S_4_ was CNY 28,214, 56,428, 84,642, and 4,213,804, respectively, and the cost of inoculation in S_1_-S_4_ gradually increased.

##### Storage and transportation of IC

Storage and transportation IC = Δvaccine cold chain cost + Δtransportation cost = ΔChange in vaccine volume × (cold chain cost per unit volume + transportation cost per unit volume).

In this study, the change ratio of the total volume of NIP vaccine for each child after the introduction of sIPV was calculated. If the change range was ±20%, the change in storage and transportation costs was considered to be 0; the judgment basis was as follows: (1) The cold chain volume matching degree (MVC) refers to the ratio of the vaccine-cold chain capacity needs of a region to the capacity of cold chain equipment required to store vaccines when providing vaccination services for the population in the region within a certain period of time. 0.8 ≤ MVC ≤ 1.2 means that it can operate normally without adding cold-chain equipment (18). (2) The Hangzhou Center for Disease Control and Prevention (CDC) entrusted the storage and transportation of vaccines in the immunization program to qualified third-party vaccine distribution units for distribution; of the 15 district and county CDCs in Hangzhou, 13 were entrusted with the storage and transportation of all immunization and non-immunization vaccines to two qualified third-party vaccine distribution units; therefore, the introduction of sIPV has little impact on the volume of vaccine storage and transportation, does not require the increase in the cost of cold chain equipment and transportation, and does not affect the price of vaccine storage and transportation services.

S_1_/S_2_/S_3_/S_4_ vaccine volume changes were calculated using the WHO-recommended vaccine volume calculation tool (19). The total volume of NIP vaccines/children was calculated according to the vaccine package volume of China's immunization program in Supplementary Table 3, the total volume of NIP vaccine/children was calculated, and the volume change rate of the PV strategy was compared. The adjustment of S_1_/S_2_/S_3_/S_4_ was 3.04–9.12% of the total volume change rate of the vaccine (Table 1). From the perspective of the society as a whole, this study considered that the overall storage and transportation cost of sIPV introduced by NIP remained unchanged; ΔStorage and transportation cost = 0.

**Table 1:** Vaccine volume calculation tool to calculate the volume change of NIP vaccine for each child.

**Strategy**	**Total volume/Child (ml)**	**Change rate (%)**
S_0_	553.69	0.00
S_1_	570.52	3.04
S_2_	587.35	6.08
S_3_	604.17	9.12
S_4_	578.93	4.56

NIP, non-immunization program.

##### Medical waste treatment IC

The medical waste treatment IC of S_1_/S_2_/S_3_/S_4_ = medical waste treatment cost of S_1_/S_2_/S_3_/S_4_-medical waste treatment cost of S_0_.

Cost of medical waste disposal = number of newborns × PV vaccination rate × weight of medical waste in each child × unit price of medical waste disposal.

Medical waste refers to the use of disposable syringes during vaccination, cotton swabs, Schering bottles, ampule bottles, etc., and the medical waste disposal management team understands the fees related to the disposal of this type of wastes. For hospitals without beds, CNY 1,000 is usually charged per month; however, as every collector uses one box only, a preferential price of 800 per month is offered, which is paid annually. Vaccination clinics were also informed that the medical waste disposal management cost for PV using different strategies would not increase. Therefore, the IC of medical waste treatment in S_1_/S_2_/S_3_/S_4_ was 0.

##### IC for adverse reactions to PV

IC for S_1_/S_2_/S_3_/S_4_ associated with the adverse reactions to PV = S_1_/S_2_/S_3_/S_4_ associated with the adverse reactions to PV cost–S_0_ associated with the adverse reactions to PV cost.

The WHO official position paper mentioned that sIPV is one of the safest vaccines in RI, whether used alone or in combination (20). There were no confirmed adverse events other than mild local erythema (0.5–1%), induration (3–11%), and tenderness (14–29%) (20). No serious adverse reactions were observed, and the cost of sIPV was set at 0 after discussion with the experts.

OPV is a safe vaccine; however, adverse events can occur in rare cases. In this study, cost referred to the cost of treating VAPP caused by OPV.

Cost of VAPP = cost of VAPP disabling + cost of VAPP dying = Number of OPV first-dose vaccinations × incidence rate of VAPP × (1-VAPP fatality rate) × cost of VAPP disability/cases + number of OPV first-dose vaccinations × incidence rate of VAPP × mortality rate of VAPP × cost of VAPP death/cases.

The incidence of VAPP varies greatly from country to country owing to the sensitivity of the monitoring systems. Platt et al. evaluated the incidence of VAPP caused by OPV use in 4.70 per million neonates worldwide (range: 2.40–9.70 per million neonates) (21). The AFP monitoring system in Hangzhou is highly sensitive, and the incidence of VAPP was 8.82/million neonates from 2010 to 2014 (22), which was higher than that reported by Platt et al., but within the credible range; therefore, the incidence of VAPP caused by OPV was set at 8.82/million neonates in this study.

No VAPP-related deaths were reported in Hangzhou from 2010 to 2014, which may be due to the low number of VAPP cases (five cases). Foreign studies conducted by Griffiths et al. set the fatality rate of VAPP at 5% (23). According to the data from the China CDC, the fatality rate of VAPP is estimated to be 2.36% (24). Combined with the local monitoring situation, the fatality rate of VAPP caused by OPV was 2.36% in this study. Ulla et al. set the VAPP failure coefficient to 0.37. Based on the research data from Li Renpeng in China, the VAPP failure coefficient was set to 0.48.

According to the monitoring results of the immune success rate in this study, the positive conversion rates of poliovirus type I antibody and poliovirus type III antibody in the one-sIPV-two-bOPV, two-sIPV-one-bOPV, and three-sIPV groups were 100%, except for the positive conversion rates of poliovirus type II antibody, which were 81, 96, and 99%, respectively. Therefore, according to the above results, the rate reduction in the incidence of VAPP after the first dose of sIPV inoculation was set to 80% in this study; after the second dose, it was set to 100%.

Cost of VAPP-disabled cases/case = (direct cost + indirect cost)/case = [(medical expenses + transportation expenses of patients in hospital + transportation and accommodation expenses of escorts) + (income reduced due to disability + government subsidies + time lost for escorts)]/case.

Cost of death from VAPP = (direct cost + indirect cost)/case = [(medical expenses + hospitalization transportation expenses + transportation and accommodation expenses of escorts) + (income reduced due to disability + government subsidies + time lost for escorts)]/case.

Dai et al. (25) investigated the disease burden of polio cases in 1993, after which no other relevant research data were available in China. This study aimed to compound the use cost data in this study, using the Net Present Value method to translate medical expenses, accommodation expenses, and transportation expenses into the present value of 2018, and using the price index R for medical care and personal goods at the discount rate (26). For the days lost in the study, the government should refer to the Zhejiang Province compensation method for abnormal reactions to vaccination: one-off compensation will be provided in case of death caused by an abnormal reaction to vaccination. The amount of economic compensation shall be calculated according to the following items and standards: 10 years for the death of urban residents age < 18 years (including those aged 18 years), calculated according to the per capita disposable income of urban residents in the previous year, and 15 years of compensation for death of those aged > 18 years: Economic compensation amount = per capita disposable income of urban residents in Zhejiang province in the previous year × compensation years. VAPP is prevalent among people aged < 18 years; therefore, the compensation period is 10 years. One-off compensation should be provided to residents with disability caused by an abnormal reaction to the vaccination. The economic compensation amount is calculated according to the following items and standards: disability grade based on the “medical accident classification standard (trial),” per capita disposable income of urban residents in Zhejiang province in the previous year, compensation period (i.e., the longest compensation is 20 years from the date of disability), and disability coefficient (1–0.1, which corresponds to disability grades 1 B to grade 3 E). The disability grade was grade 3B, the disability coefficient was 0.4, and the compensation period was calculated based on a 20-year period:

Medical expenses/case = Medical expenses × $\prod_{1993year}^{2018year}\left(1+r\right)$

Inpatient transportation cost/case = inpatient transportation cost (25) × $\prod_{1993year}^{2018year}\left(1+r\right)$

Transportation and accommodation costs of attendants/case = transportation and accommodation costs of attendants (25) × $\prod_{1993year}^{2018year}\left(1+r\right)$

Attendant missing work = number of days attendants missed work (25) × per capita GDP in 2018/365 days.

Income lost due to disability = 2018 GDP per capita × 30 years of service × VAPP disability coefficient.

According to the above formula, the burden of VAPP-disabling diseases is CNY 3,211,803.00, and the burden of fatal VAPP diseases is CNY 5,616,005.49 (Table 2).

**Table 2:** Disease burden of VAPP disability and fatality cases.

**Type**	**Cost type**	**Items**	**Cost** **(CNY/case)**	**Time (Day/case)**	**Total cost** **(CNY/case)**
Disability	Direct cost	Medical	1,654.4	0	3,420.27
Disability	Direct cost	Hospital transportation costs for patients	461.48	0	954.05
Disability	Direct cost	Hospital transportation costs for attendants	306.39	0	633.42
Disability	Indirect cost	Attendants miss work	384.05	173.84	66,763.25
Disability	Indirect cost	Loss of income due to death	0	0	2,018,592.00
Disability	Indirect cost	The government compensation	0	0	1,121,440.00
VAPP disability disease burden			0	0	3,211,803.00
Fatality	Direct cost	Medical	479.74	0	991.80
Fatality	Direct cost	Hospital transportation costs for patients	799.18	0	1,652.21
Fatality	Direct cost	Hospital transportation costs for Attendants	398.18	0	823.19
Fatality	Indirect cost	Attendants miss work	384.05	13.90	5,338.30
Fatality	Indirect cost	Loss of income due to death	0	0	4,205,400.00
Fatality	Indirect cost	The government compensation	0	0	1,401,800.00
VAPP fatality disease burden			0	0	5,616,005.49

VAPP, vaccine-associated paralytic poliomyelitis; CNY, Chinese Yuan.

In S_0_, the average annual disease burden of VAPP was CNY 704,219.34. In S_1_/S_2_/S_3_, more than 2 doses of sIPV were administered, no VAPP occurred, and the disease burden of VAPP was CNY 0. In S_4_, 50% of the population was vaccinated with only 1 dose of sIPV. Hence, the average annual disease burden of S_4_ VAPP was CNY 352,109.67. The disposal of adverse reactions after S_1_/S_2_/S_3_/S_4_ PV inoculation were as follows: CNY −704,219.34, −704,219.34, −704,219.34, and −352,109.67, respectively.

##### VDPV-Related IC

VDPV-related IC of S_1_/S_2_/S_3_/S_4_ = VDPV-related cost of S_1_/S_2_/S_3_/S_4_-VDPV-related cost of S_0_.

VDPV-related cost =circulating vaccine-derived viruses (cVDPV) cost + immunodeficiency vaccine-derivcd poliovirus (iVDPV) cost + ambiguous vaccine-derivcd poliovirus (aVDPV) cost.

According to surveys (27–29), no aVDPV was found in China and Hangzhou, so the aVDPV cost was regarded as zero in this paper.

###### cVDPV cost

Globally, an average of 76 cVDPV cases occur each year, mainly in 23 less developed countries (30). Considering the previous cVDPV incidence in China, cVDPV I occurred in Zhenfeng County, Buyi, and Miao Autonomous Prefecture, Guizhou Province in 2004, and two cases of VDPV I were reported. A circulating polio-hypervariable virus was detected in Aba County, Aba Tibetan, and Qiang Autonomous Prefecture, Sichuan Province in 2012. One case of cVDPV II and three cases of VDPV II were reported. The national cVDPV incidence is one case every 5 years, with three polio cases per case. According to a comparison between the population of Hangzhou and the national population, the number of cVDPV cases per year was 0.01. For reference, the cost of emergency work after the occurrence of cVDPVs in Guizhou province in August 2004 = the cost of OPV enhanced immunization + the cost of fecal specimen collection, transportation, and testing = CNY 52.85 million (24); the average annual cost of emergency work caused by cVDPV in Hangzhou is CNY 2,114 million. Disease burden of polio cases = 0.004 cases × (disease burden of VAPP disability cases × (1-VAPP fatality rate) + disease burden of VAPP fatality cases × VAPP fatality rate). The average annual burden of cVDPV in China was CNY 12,597.03. The cVDPV incidence of S_0_/S_1_/S_2_/S_4_ is the same, the cVDPV cost of S_0_/S_1_/S_2_/S_4_ is CNY 223,977.03, and the cVDPV incidence and related costs of S_3_ are 0. The annual cVDPV-related IC of S_1_/S_2_/S_3_/S_4_ were as follows: CNY 0, 0, −223,977.03, and 0.

###### iVDPV cost

One case of iVDPV was reported in Anhui in 2005, Ningxia in 2011, Tianjin in 2012, and Jiangxi in 2013 (31), while one iVDPV case was reported every 2 years in China. According to the results of the comparison between the population of Hangzhou and the national population, the annual iVDPV of Hangzhou is 0.0033. Based on the cost of carrying out emergency immunization and case surveillance after the occurrence of iVDPVs in Anhui province in 2005, iVDPV emergency work cost = OPV emergency immunization cost + stool specimen collection, transportation, and testing cost + construction of sanitary toilets + disinfection cost + personnel subsidy = CNY 4.49 million/case. The disease burden of iVDPV cases is regarded as VAPP fatality cases = CNY 5,616,005.49/case. Before OPV use is terminated, the risk of iVDPV always exists. The incidence of iVDPV of S_0_/S_1_/S_2_/S_4_ is the same. The iVDPV cost of S_0_/S_1_/S_2_/S_4_ = (CNY 4.488 million/case +CNY 5,616,005.49/case) × 0.0033 = CNY 33,343.22/year. The incidence and cost of iVDPV in S_3_ were zero, while the annual iVDPV-related ICs of S_1_/S_2_/S_3_/S_4_ were CNY 0, 0, −33,343.22, and 0, respectively.

The annual VDPV-related ICs of S_1_/S_2_/S_3_/S_4_ were CNY 0, 0, −257, 320.25, and 0, respectively.

##### Traffic cost and IC of missed work for parents taking their children for vaccination

The cost of traffic and cost of delayed work only had an impact on S_4_. In the immunization program, OPV was administered at the same time at 3, 4, and 18 months of age, and 50% of children in S_4_ went to the vaccination clinic one less time compared with those in S_0_. Traffic cost and time cost of parents per inoculation = estimated traffic cost per inoculation + number of accompanying personnel × number of missed work days × per capita GDP in 2018/365 days = CNY 20/time +1.5 accompanying personnel/time × 0.5 days × per capita GDP in 2018/365 = CNY 308.04/time. The total cost = number of newborns in 2018 × PV coverage rate × 0.5, doses × traffic cost and time cost of parents per vaccination = CNY 11,811,890.36. In S_1_/S_2_/S_3_/S_4_, the traffic and delayed work costs of parents taking their children to vaccinate were CNY 0, 0, 0, and −11,811,890.36, respectively.

### Sensitivity analysis

A univariate sensitivity analysis was conducted to explore the impact of parameter uncertainty by considering simultaneous changes within plausible ranges. The incidence of VAPP cases with the five schedules, compensation cost of VAPP cases, and cost of IPV were included according to the range of values to evaluate the influence of changes in these parameters on the ICER.

## Results

### Incremental cost

The proposed variable cost composition of this study includes 7 items, while the ICs of S_1_/S_2_/S_3_/S_4_ are CNY 30.77, 68.58, 103.82, and 219.82 million, respectively (Table 3).

**Table 3:** IC of S_1_/S_2_/S_3_/S_4_ (CNY).

**Strategy**	**Vaccine IC**	**Vaccination IC**	**Storage and transportation IC**	**Medical waste treatment IC**	**Adverse reactions to PV vaccination IC**	**VDPV related IC**	**Parents traffic delay IC**	**IC**
S_1_	3,752,851	28,214	0	0	−704,219.34	0	0	3,076,845.66
S_2_	7,505,703	56,428	0	0	−704,219.34	0	0	6,857,911.66
S_3_	11,258,554	84,642	0	0	−704,219.34	−257320.25	0	10,381,656.41
S_4_	29,931,938	4,213,804	0	0	−352,109.67	0	−11,811,890	21,981,742.33

IC, incremental cost; CNY, Chinese Yuan; VDPV, vaccine-derived poliovirus.

### Incremental effectiveness

S_1_/S_2_/S_3_/S_4_ IE_VAPP_ = S_0_ annual number of VAPP cases—S_1_/S_2_/S_3_/S_4_ annual number of VAPP cases.

IE_DALY_ of S_1_/S_2_/S_3_/S_4_ = annual DALY lost to VAPP cases in S_0_–annual DALY lost to VAPP cases in S_1_/S_2_/S_3_/S_4_.

DALY loss caused by VAPP = (number of VAPP disability cases × disability coefficient + number of VAPP fatality cases) × average life expectancy.

In S_0_, the number of VAPP cases per case was 0.22; in S_4_, the number of VAPP cases per case was 0.11; and in S_1_/S_2_/S_3_, no VAPP cases were reported.

The IE_VAPP_ cases for S_1_/S_2_/S_3_/S_4_ were 0.22, 0.22, 0.22, and 0.11, respectively.

The IE_DALY_ of S_1_/S_2_/S_3_/S_4_ were 8.98, 8.98, 8.98, and 4.49, respectively.

### Incremental cost-effectiveness ratio

The ICER_VAPP_ of S_1_/S_2_/S_3_/S_4_ increased gradually to CNY 13.99, 31.17, 47.19, 199.83 million/VAPP, respectively. The ICER_DALY_ of S_1_/S_2_/S_3_/S_4_ also increased gradually, which were CNY 0.34, 0.76, 1.16, and 4.90 million/DALY, respectively (Table 4).

**Table 4:** ICER of S_1_/S_2_/S_3_/S_4_.

**Strategy**	**Reduction of VAPP cases (case)**	**ICER_VAPP_ (CNY, million)**	**Reduction of DALY cases (case)**	**ICER_DALY_ (CNY, million)**
S_1_	0.22	13.99	8.98	0.34
S_2_	0.22	31.17	8.98	0.76
S_3_	0.22	47.19	8.98	1.16
S_4_	0.11	199.83	4.49	4.90

ICER, incremental cost-effectiveness ratio; VAPP, vaccine-associated paralytic poliomyelitis; CNY, Chinese Yuan; DALY, disability-adjusted life-years.

ICER_VAPP_ and ICER_DALY_ were substantially high for S_3_ (four-sIPV) and S_4_ (replacement of self-funded sIPV based on one-sIPV-three-bOPV). The recommendations of WHO on pharmacoeconomic evaluation (32): ICER_DALY_ < per capita GDP, with high cost-effectiveness; per capita GDP < ICER_DALY_ < 3 times GDP per capita, cost-effectiveness; and ICER_DALY_ >3 times per capita GDP, without cost-effectiveness advantage. In 2018, the per capita GDP of the Hangzhou Statistics Bureau was CNY 0.14 million/year. S_1_ (two-sIPV-two-bOPV) had a cost-effectiveness advantage, whereas S_2_/S_3_/S_4_ had no cost-effectiveness advantage.

## Discussion

The economic benefits of IPV-containing immunization schedules need to be further clarified. This study evaluated the cost-effectiveness of different IPV-containing immunization schedules in Hangzhou, which could provide convincing evidence to further switch PV immunization strategies in China. In this study, we found that two-sIPV-two-bOPV has a cost-effectiveness advantage, whereas other schedules have no cost-effectiveness advantage. IPV-containing schedules are currently cost-effective in Hangzhou. They may be cost-effective by reducing the prices of sIPV, which may accelerate polio eradication in China.

Recent studies have shown that sIPV inclusion in the immunization schedule remains cost-effectiveness, although it differs extensively between studies (33–36). Studies have shown that Global OPV cessation offered the possibility of large future health and economic benefits compared with continued OPV use (37). OPV remains the PV of choice in most countries because of their low cost (38). Most high-income countries use IPV exclusively for RI, while middle-income countries continue to adopt IPV for RI using a sequential schedule of IPV followed by OPV (IPV/OPV) or an IPV dose co-administered with the third non-birth OPV dose (38). IPV remains much more expensive than OPV but does not come with VAPP or cVDPV risks because it does not contain a live poliovirus (39). In upper-middle-income countries, PV immunization schedules are cost-effective in addition to their efficacy in preventing diseases, which means that the expected cost of treatment exceeds the difference in the cost between the schedules (40), with the best estimates suggest incremental net benefits for the intervention of between ~$40 and $50 billion when compared to Routine vaccination, assuming reasonable economic values for the prevention or treatment of paralysis (40). By contrast, in low-income countries, the cost of immunization schedules remains relatively high and the treatment cost is much lower, which implies the need to pay additional costs to obtain effectiveness (40). In addition, projections of the cost of the vaccine are greatly dependent on supply and the ability to negotiate a competitive price (17). Estimates of vaccine costs were largely driven by the assumption that middle-income countries would gradually switch to IPV in any scenario. With the large population of middle-income countries, including China, and the higher cost of IPV, middle-income countries contribute significantly to the difference in the global cost of the different policy scenarios. If the price of IPV decreased over time or if fewer middle-income countries were to switch to IPV, the cost of universal IPV would be lower. In this study, ICER_DALY_ of S3 (4 doses sIPV) = 1.16 million, had no cost-effectiveness advantage. If 0.14 million < ICER_DALY_ of S3(4 doses sIPV) < 0.42 million, S3 would had a cost-effectiveness advantage. Assuming everything else is equal, the price for sIPV is between CNY 10 to CNY 17 could make 4 doses sIPV schedule have cost-effectiveness advantage. Vaccine costs are not the only factor to be considered in policy decision-making. This suggests the possible limitations of utilizing the ICER in vaccine evaluation. The fundamental issues that should be taken into account when deciding to introduce a new vaccine include the type of vaccine, the target disease, and the strength of immunization program (41).

Policymakers should consider not only the costs of global policies but also potential benefits such as prevention of VAPP cases. The risk of VAPP continues to exist for policies that involve OPV use, and the continuous use OPV increases the number of VAPP cases. Findings from the health economics evaluation on replacing OPV with IPV varied widely: ICER_VAPP_ ranged between USD 0.74 and 80.0 million, while ICER_DALY_ caused by VAPP ranged between USD 0.01 and 5.60 million (37, 42). If the introduction of sIPV doses is associated with the replacement of the respective OPV doses, the cost increase will be approximately USD 20 and 59 million, respectively (35). Replacing the first three OPV doses with IPV on RI with discontinuation of supplemental immunization reduced the incidence of VAPP to 51 (35). In this study, we found that ICER_VAPP_ and ICER_DALY_ were substantially high for four-sIPV (CNY 47.19 million and 1.16 million), and the replacement of self-funded sIPV based on one-sIPV-three-bOPV (CNY 199.83 and 4.90 million), compared with those of the previous one-sIPV-three-bOPV schedule in < city>Hangzhou < /city>, China. Similar results were reported in previous studies. The two-IPV-two-bOPV and four-IPV schedules could prevent 1.20–1.35 VAPP cases and avert 16.83–18.96 DALYs per year, compared with the four-tOPV schedule in Shanghai, China (43). In Russia, the incidence of VAPP cases was much lower for sequential immunization schedule of OPV and IPV (1 case/4.18 million doses) than that for the OPV-only schedule (1 case/1.59 million OPV doses) (44). In the United States, no VAPP occurred in the IPV-containing schedule, while 2.9 VAPP cases/million occurred in the OPV schedule (45). ICER_VAPP_ and averting DALY_VAPP_ varied widely (40, 42, 46). In this study, the two-sIPV-two-bOPV immunization schedule was cost-effective, according to the WHO guidelines (47). Our findings suggest that the lower cost of sIPV and higher VAPP incidence may contribute to the lower ICER and higher cost-effectiveness. The ICER varies across countries with different economic status (33, 42). In 1996, an American study of children aged < 6 years reported an ICER_VAPP_ of USD 3.1 million per VAPP for the two-IPV-two-bOPV schedule and USD 3 million per VAPP for the four-IPV schedule (46). By contrast, a 2001 Australian study conducted in children aged < 6 years showed that the ICER_VAPP_ were USD 10 million per VAPP and USD 29 million per VAPP for these two schedules (48). In addition, the ICER_DALY_ in Columbia was USD 0.071 million per DALY (42). The disparity in ICER_VAPP_ across countries may be related to the differing costs of IPV and OPV, incidence of VAPP, and compensation costs for VAPP in different countries (33–36).

However, IPV does not protect against infections or from participation in asymptomatic fecal-oral poliovirus transmission, and its ability to stop or prevent poliovirus transmission were not tested in developing countries (39, 49). Consistent with the data from clinical trials showing limited intestinal immunity provided by IPV (49), despite an IPV-only RI coverage of over 90%, Israel recently detected intense asymptomatic WPV1 transmission for 12 months, likely due to the relatively low hygienic conditions in the Bedouin populations in the South (50, 51). IPV may offer a relatively greater reduction in long-term global risks associated with iVDPV introduction or other releases and may help prevent sustained transmission of OPV-related viruses and thus cVDPV emergence in settings with higher RI coverage and less fecal-oral transmission (52). Although the plan anticipates the simultaneous globally coordinated cessation of serotype 1-containing OPV (OPV1) and serotype 3-containing OPV (OPV3) after 2018 (OPV13 cessation), the possibility of certification of global WPV3 interruption in 2016, while WPV1 may continue to circulate, raising the potential for phased withdrawal of OPV3 and then OPV1 (30). Duintjer's study showed that IPV may prevent initial transmissions that lead to the establishment of population-wide transmission (37). No viable outbreak response strategy exists to stop poliovirus spread if it occurs more than 5–10 years after OPV cessation and in the absence of a large mOPV stockpile in populations highly susceptible to fecal-oral poliovirus transmission. The cost of maintaining an outbreak response capacity represents about 10% of the total program cost. However, an increase in the price of OPV would increase the cost of maintaining stockpiles and responding to outbreaks. This analysis aims to show policymakers the relative costs associated with various sIPV immunization policies with different doses. This analysis presents some of the costs including vaccine IC, vaccination IC, adverse reactions to PV vaccination IC, VDPV related IC, and parents traffic delay IC that will affect the IC of sIPV immunization program with different doses. The four PV immunization schedules in this study have both advantages and disadvantages in terms of safety, effectiveness, accessibility, and cost of administration (46, 53). To achieve the goal of polio eradication, VAPP and VDPV caused by OPV use should be eliminated (54). IPV provides protection against all three poliovirus serotypes, which facilitates the maintenance of polio-free status (55). Therefore, the possible social and economic impact of VAPP cannot ignore, such as the vaccine hesitancy in Nigeria in 2003 (56), although the four-IPV schedule was cost-ineffective in our study. In Shanghai in 2019, in the context of implementing the free two-IPV-two-bOPV schedule, 47% of children's parents chose the four-IPV schedule at their own expense or an IPV-containing combined vaccine (unpublished data), suggesting a high intention to receive IPV. This report indicates the feasibility of including the four-IPV immunization schedule in local EPI in resource-rich Chinese settings, such as the metropolitan areas like Shanghai (43). Other key uncertainties that affect the probability of outbreaks and/or their consequences include the long-term survival of immunodeficient patients with lower income levels, the impact of IPV-induced immunity on transmission and/or extent of fecal-oral spread in different populations, the quality and frequency of tOPV rounds until OPV2 cessation and bOPV rounds leading up to OPV13 cessation, the future rate of release of WPV or sIPV production sites in the context of different levels of containment, the unpredictable occurrence of other extremely rare events after OPV cessation with very large consequences, and the potential for OPV used during outbreak response to generate new VDPV outbreaks elsewhere (53, 57).

As with any model, this study had some limitations. Our analysis did not consider all the costs. For example, we did not estimate the expected cost of an outbreak; doing so would entail the generation of values for the risks of political re-emergence. However, one would anticipate that the costs of responding to an outbreak would include a mass immunization response, increased surveillance and case investigation, training of responders, and replenishing the stockpile. The management of these risks and the expected costs and benefits will ultimately drive policy decisions. The cost of cold-chain services, human resources, administration cost of human resources, and administration of VAPP surveillance, treatment, follow-up, and compensation are difficult to accurately estimate. Thus, we hypothesized that they would remain identical across the four PV immunization schedules. However, this study provided better estimations based on previous findings. Further studies will need to consider the full set of prospective vaccines and other policy options, and the impacts of immunization policy decisions on expected future polio endgame health and economic costs.

## Conclusion

This study found that ICER_VAPP_ and ICER_DALY_ were substantially high for S_3_ (four-sIPV) and S_4_ (replacement of self-funded sIPV based on one-sIPV-three-bOPV). Two-sIPV-two-bOPV had a cost-effectiveness advantage, whereas S2/S3/S4 had no cost-effectiveness advantage, which might be attributable to the difference in the cost between sIPV and OPV and rare VAPP cases. However, they may be cost-effective by decreasing sIPV prices. Considering the risk of VAPP in the four-tOPV schedule, IPV-containing schedules may be a feasible strategy to accelerate polio eradication efforts in China. In addition, further evaluation of the long-term effectiveness and benefits of IPV-containing schedules in China is warranted.

## Data availability statement

The original contributions presented in the study are included in the article/Supplementary material, further inquiries can be directed to the corresponding author.

## Author contributions

YX and YL: formal analysis and methodology. JW, XC, XuZ, and WJ: investigation. YL: project administration and writing—review and editing. YX: software and writing—original draft. JD, XiZ, and WG: validation. All authors contributed to the article and approved the submitted version.

## Funding

This study was funded by the Zhejiang Provincial Basic Public Welfare Research Projects (Grant No. LGF18H260002) and Hangzhou Research Projects for Agricultural and Social Development (Grant No. 20180533B94).

## Conflict of interest

The authors declare that the research was conducted in the absence of any commercial or financial relationships that could be construed as a potential conflict of interest.

## Publisher's note

All claims expressed in this article are solely those of the authors and do not necessarily represent those of their affiliated organizations, or those of the publisher, the editors and the reviewers. Any product that may be evaluated in this article, or claim that may be made by its manufacturer, is not guaranteed or endorsed by the publisher.
