# Supplementary material for: Cost-effectiveness of various immunization schedules with inactivated Sabin strain polio vaccine in Hangzhou, China
Source: Front Public Health. 2022 Sep 23;10:990042. doi: 10.3389/fpubh.2022.990042 (PMC9545176; doi:10.3389/fpubh.2022.990042)
Supplement: Supplementary file 1 [file Data_Sheet_1.pdf]

Supplementary Table 1 Total population, birth rate and number of newborns in Hangzhou from 2013 to 2018

Year	Total population	Birth rate of newborns（‰）	Number of newborns
2013	9368416	11.55	108202
2014	9413631	13.85	130395
2015	8484541	12.24	103882
2016	9432506	14.82	139819
2017	9544209	14.31	136574
2018	9832461	12.34	121365
Average	9345961	13.20	123373

Supplementary Table 2 Use of PV in Hangzhou from 2016 to 2018

Year	first needle	second needle	third needle	fourth needle	IPV (Self-funded)	DTaP/Hib/IPV conjugate vaccine	IPV(free)	bOPV	Replacement rate of Self-funded IPV(%)
2016	131209	131247	129295	162268	101578	153268	57109	242064	51.29
2017	150418	154854	159008	121545	19695	142197	118579	305354	34.65
2018	126510	130018	134510	119713	469	72119	135929	302234	19.37
Total	408137	416119	422813	403526	121742	367584	311617	849652	36.54

Acronyms:PV=poliovirus vaccine;IPV=inactivated poliovirus vaccine;bOPV=bivalent attenuated oral poliovirus vaccine.

Supplementary Table 3 List of vaccine packages in the immunization programme

Vaccines	Approval Number	Manufacturer	Packaging Specification	Unit Packaging Volume (cm^3)
Sabin strain inactivated poliomyelitis vaccine (Vero cell)	20170006	Beijing Institute of Biological Products Co., LTD.	3 PCS in a box	60.10
Sabin strain inactivated poliomyelitis vaccine (Vero cell)	20150002	Institute of Medical Biology, Chinese Academy of Medical Sciences	1 piece in a box	39.01
Oral type I, Ⅲ poliomyelitis live attenuated vaccine (human diploid cell)	20150014	Beijing Institute of Biological Products Co., LTD	3 PCS in a box	60.10
Recombinant hepatitis B vaccine (Hanson's yeast)	20040016	Aimei Hissen Vaccine (Dalian) Co., LTD	30 PCS in a box, no needle	380.8
Lyophilized live attenuated hepatitis A vaccine	20000063	Zhejiang Pukang Biotechnology Co., LTD	1 piece in a box	72.8
BCG for intradermal injection	20013057	Chengdu Institute of Biological Products Co., LTD	10 PCS in a box	252.8
Adsorbed Acellular DPT combined vaccine	10970013	Chengdu Institute of Biological Products Co., LTD	3 PCS in a box	84.59
MMR combined live attenuated vaccine	20020108	Beijing Institute of Biological Products Co., LTD	1 piece in a box	58.97
Adsorbed diphtheria tetanus combined vaccine	10820043	Wuhan Institute of Biological Products Co., LTD.	5 PCS in a box	138.84
Live attenuated Japanese encephalitis vaccine	19980008	Chengdu Institute of Biological Products Co., LTD	1 piece in a box	39.69
Meningococcal polysaccharide vaccine group A	10820032	Wuhan Institute of Biological Products Co., LTD.	5 PCS in a box	73.37
Meningococcal polysaccharide vaccine group A+C	20010092	Lanzhou Institute of Biological Products Co., LTD.	5 PCS in a box	95.26

Acronyms:BCG=bacillus Calmette-Guérin;DPT=Diphtheria, Tetanus,Pertussis combined vaccine;MMR=Live attenuated measles, rubella and mumps vaccine;Co., LTD.=Company Limited;PCS=Pieces.
